# Emerging dilemmas in the diagnosis and management of gastroesophageal reflux disease

**DOI:** 10.12688/f1000research.11918.1

**Published:** 2017-09-25

**Authors:** Peter Kahrilas, Rena Yadlapati, Sabine Roman

**Affiliations:** 1 Department of Medicine, Feinberg School of Medicine, Northwestern University, 676 St. Clair Street, 14th floor, Chicago, IL, 60611-2951, USA; 2 Digestive Physiology, Hospices Civils de Lyon and Lyon I University, Lyon, France

**Keywords:** GERD, PPI

## Abstract

Gastroesophageal reflux disease (GERD) is common, but less so than widely reported because of inconsistencies in definition. In clinical practice, the diagnosis is usually based on a symptom assessment without testing, and the extent of diagnostic testing pursued should be limited to that which guides management or which protects the patient from the risks of a potentially morbid treatment or an undetected early (or imminent) esophageal adenocarcinoma or which does both. When testing is pursued, upper gastrointestinal endoscopy is the most useful initial diagnostic test because it evaluates for the major potential morbidities (Barrett’s, stricture, and cancer) associated with GERD and facilitates the identification of some alternative diagnostic possibilities such as eosinophilic esophagitis. However, endoscopy is insensitive for diagnosing GERD because most patients with GERD have non-erosive reflux disease, a persistent diagnostic dilemma. Although many studies have tried to objectify the diagnosis of GERD with improved technology, this is ultimately a pragmatic diagnosis based on response to proton pump inhibitor (PPI) therapy, and, in the end, response to PPI therapy becomes the major indication for continued PPI therapy. Conversely, in the absence of objective criteria for GERD and the absence of apparent clinical benefit, PPI therapy is not indicated and should be discontinued. PPIs are well tolerated and safe, but nothing is perfectly safe, and in the absence of measurable benefit, even a miniscule risk dominates the risk-benefit assessment.

Gastroesophageal reflux is a normal physiological event that commonly occurs during and after meals. However, gastroesophageal reflux disease (GERD) is a “condition that develops when the reflux of stomach contents causes troublesome symptoms and/or complications” ^1^. This umbrella definition was devised to encompass the broad spectrum of GERD, inclusive of endoscopically evident disease (esophagitis, stricture, Barrett’s metaplasia, and adenocarcinoma), troublesome esophageal symptoms without endoscopically evident disease (heartburn, regurgitation, and chest pain), and potential extra-esophageal manifestations such as laryngitis or cough.

Although the above definition of reflux disease, widely referred to as the Montreal definition, was intellectually satisfying in fusing the potential manifestations of GERD by the common element of stemming from the reflux of gastric content into the esophagus, it also posed some significant practical problems for the clinician. Notable among these are (1) how to establish causality between inherently non-specific symptoms and reflux and (2) defining the threshold frequency or severity at which a symptom becomes “problematic”. Grappling with these issues has fostered an environment in which the over-diagnosis and over-treatment of GERD have become rampant. That, in turn, has led to the substantial overuse of GERD treatments, especially proton pump inhibitors (PPIs). It is from this background that we will address the following emerging dilemmas of managing reflux in 2017: (1) How is GERD defined? (2) How is GERD diagnosed? And (3) what are the indications and risks for long-term PPI therapy?

## How is gastroesophageal reflux disease defined?

**Figure 1.**
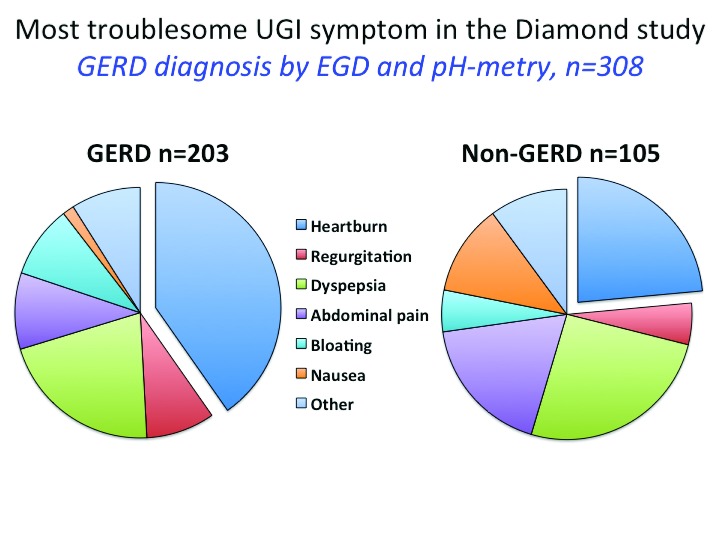
Symptom profiles of patients with and without objective evidence of gastroesophageal reflux disease (GERD) (upper gastrointestinal (GI) endoscopy or pH-metry or both) in the Diamond study ^2^. The entry criterion for the multinational primary care study was the presence of a troublesome upper GI symptom. Plotted here were the dominant symptoms reported by each participant. All subjects were studied with endoscopy and pH-metry and underwent a structured interview with both a general practitioner and a gastroenterologist.

The typical symptoms of GERD are heartburn (a burning sensation arising behind the breastbone toward the neck) and regurgitation (experienced as refluxed fluid moving in the chest or a bitter taste in the mouth). However, these typical symptoms are neither sensitive nor specific for GERD as demonstrated in the Diamond study ^2^. In that study, 308 patients identified in primary care as having troublesome upper gastrointestinal (GI) symptoms underwent a comprehensive evaluation with endoscopy, esophageal pH-metry, structured physician interviews, questionnaires, and a trial of PPIs, thereby allowing comparisons among these diagnostic methods. When endoscopy or pH-metry was used as the diagnostic standard, 203 (66%) of these patients had GERD, but as shown in Figure 1, only a minority of the patients with GERD had heartburn or regurgitation as their dominant symptom and more than a quarter of those without GERD indicated one of these as their dominant symptom. Not surprising then was the finding that the sensitivity and specificity of the Reflux Disease Questionnaire (RDQ) and the physician assessments were all in the range of 63% and 67%, respectively, against that same diagnostic standard.

**Figure 2.**
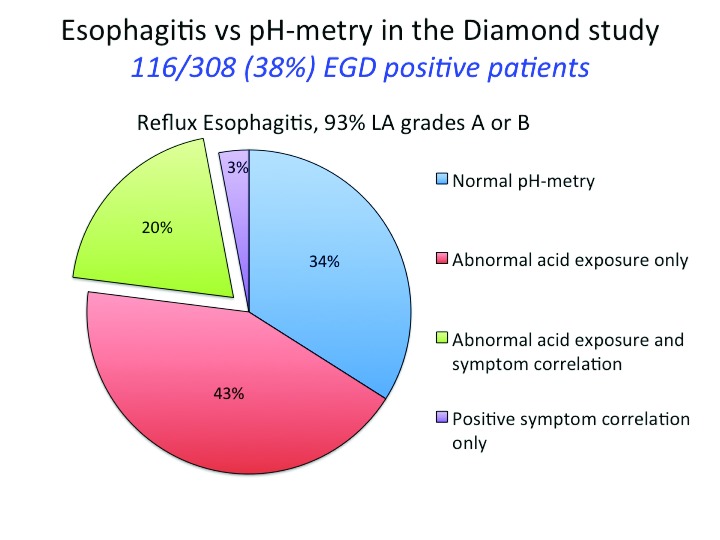
pH-metry findings among patients found to have reflux esophagitis on endoscopy in the Diamond study ^3^. pH-metry was carried out by using the wireless Bravo system, and a single 24-hour period was analyzed. Studies were interpreted as positive or negative on the basis of both esophageal acid exposure (>5%) and a positive symptom association probability score for their dominant upper gastrointestinal (GI) symptom.

The Diamond study also contrasted the diagnostic assignment made by endoscopy, pH-metry, and response to PPI therapy. Figure 2 illustrates the comparison between endoscopy and pH-metry. Note that only 20% of the patients with esophagitis had both abnormal esophageal acid exposure and a positive symptom correlation during their wireless Bravo pH-metry study and that 34% had completely normal studies. Response to a 2-week trial of esomeprazole 40 mg did not clarify these discrepancies. Even though a beneficial PPI response, defined as absence of the dominant symptom for the final 3 days of the trial, was more frequent in patients with esophagitis (69%) and in patients with non-erosive reflux disease (NERD) (49%), 35% of patients with normal endoscopy and pH-metry also had a beneficial response ^3^.

Evidently, GERD is not so easily defined. The diagnosis is variably based on endoscopy, pH-metry, symptom assessment, or response to PPI therapy, but each of these modalities identifies a distinct patient population. Furthermore, although the severity of esophagitis correlates with the extent of esophageal acid exposure during pH-metry to some degree, the same relationship does not hold for reflux symptom severity. Clearly, the pathophysiological determinants of reflux symptoms are somewhat distinct from those of mucosal erosion. Mucosal injury is facilitated by prolonged exposure to refluxed acid, pepsin, and bile acids. This is largely facilitated by an incompetent sphincter, hiatus hernia, and poor peristaltic function ^4, 5^. Symptoms, on the other hand, are strongly modulated by sensitivity. Only about 10% of reflux episodes are perceived ^6^, and patients with GERD are more sensitive to esophageal stimuli than are control subjects ^7^. Reflux episodes during which the refluxate reaches the proximal esophagus (more common among patients with GERD) are also more likely to be symptomatic, and recent physiological data suggest that the proximal esophagus has distinct innervation compared with the distal esophagus ^8^. Finally, the phenomena of hypersensitivity and hypervigilance are increasingly recognized as major determinants of symptom perception and severity among subsets of patients with NERD ^9^.

In regard to the opening question, there has been no standardized way of defining GERD. Rather, investigators and clinicians have used definitions of convenience for the task at hand, be that research, a treatment trial, or a patient encounter. True, most such definitions will fit under the umbrella of the Montreal definition, but without further qualifications, the Montreal definition is too broad for most purposes. Furthermore, since the criteria used to define GERD in epidemiological studies (for example, self-reporting of at least weekly heartburn or regurgitation or both) are even more permissive than the Montreal definition, currently reported GERD prevalence rates ranging from 9% to 33% ^10^ are likely to be over-estimates.

## How is gastroesophageal reflux disease diagnosed?

GERD is usually a clinical diagnosis based on a symptom assessment. Testing is reserved for cases in which there are warning signs of complication (dysphagia, odynophagia, weight loss, bleeding, or anemia), atypical symptoms such that the diagnosis is uncertain, an inadequate response to medical treatment, or as a preoperative evaluation to confirm excessive reflux prior to surgical treatment. Hence, the management approach used varies greatly depending on a symptom assessment, an assessment of the risk that complications exist, the history and success of treatment trials, whether or not a potentially morbid therapy is under consideration, and the history of prior testing. As a general rule, the extent of diagnostic testing should be limited to that which guides management decisions or which protects the patient from the risk of an inappropriate treatment or an undetected early (or imminent) cancer or which does both.

### Symptom assessment and questionnaires

In clinical practice, the complaint of heartburn or acid regurgitation (or both) in a patient without signs of complications is sufficient to initiate anti-reflux therapy. Questionnaires have been devised to standardize the assessment of these symptoms in order to facilitate screening for GERD in primary care settings. Bolier *et al*. recently reviewed 39 questionnaires to assess GERD symptoms, 14 to assess treatment response, and 18 to assess GERD-related quality of life ^11^. Among these, the RDQ—consisting of six items that assess the frequency and severity of heartburn, regurgitation, and dyspepsia—is one of the most widely used. The accuracy with which questionnaires diagnose GERD varies with what is used as the reference standard. If the comparison is with the diagnosis rendered by an experienced clinician, the correspondence is very good ^2^; if the comparison is with pH-metry, endoscopy, or response to PPI therapy, the sensitivity and specificity are only about 65% ^12^.

### Upper gastrointestinal endoscopy

Upper GI endoscopy is the most useful diagnostic test in GERD management. Potential endoscopic findings that might direct management include reflux esophagitis, eosinophilic esophagitis, hiatus hernia, peptic ulcer, bleeding, stricture, Barrett’s esophagus, and esophageal adenocarcinoma. With respect to diagnosing esophagitis, the minimal endoscopic lesion with acceptable inter-observer agreement is a mucosal break, the basis for the Los Angeles classification. A mucosal break is defined as “an area of slough or erythema with a discrete line of demarcation from the adjacent, more normal looking mucosa” ^13^. Within the Los Angeles scheme, the severity of esophagitis is graded A (minimal) through D (very severe) depending on the extent of the mucosal breaks observed ^13^. However, NERD is the dominant form of GERD, and esophagitis will be absent in about 70% of cases being evaluated for reflux symptoms ^14^. Consistent with this estimate, esophagitis was reported in only 17.3% of 280,075 endoscopies in the Clinical Outcomes Research Initiative database and among these, 79% were classed as mild and graded as Los Angeles A or B when Los Angeles grading was reported ^15^. Attempts to extend the diagnostic sensitivity of endoscopy to microscopic lesions more subtle than mucosal breaks have applied techniques such as magnification endoscopy with a narrow band imaging light source ^16^. However, within that context, it is important to note the finding of Los Angeles grade A esophagitis in 5% of asymptomatic controls participating in a population-based endoscopy study ^17^, leading some to question the significance of this (macroscopic) finding. Similarly, histologic examination of mucosal biopsies might increase the sensitivity for detecting GERD, but again at the expense of specificity. Microscopic esophagitis (basal cell hyperplasia, papillary elongation, dilated intercellular spaces, and inflammation) was observed in 65% of patients with NERD but also in 15% of controls ^18, 19^. In summary, endoscopy is an important test to detect esophagitis, complications of GERD, and alternative diagnoses that might redirect therapy, but it has very poor sensitivity for diagnosing GERD.

### Proton pump inhibitor trial

**Figure 3.**
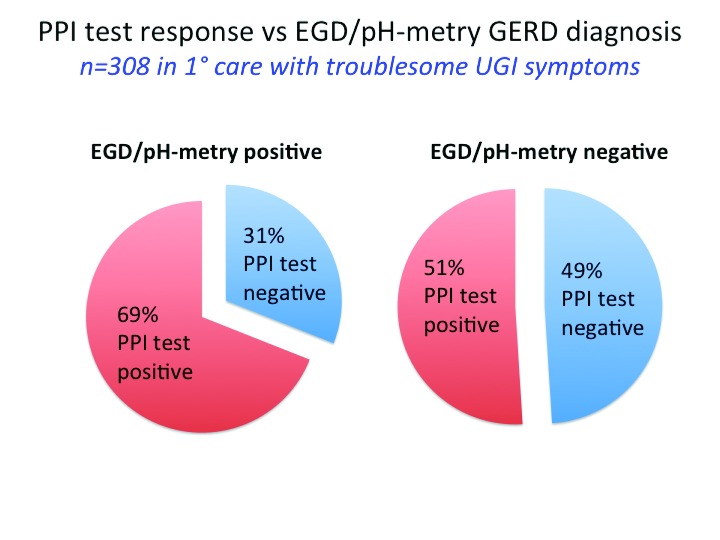
pH-metry findings versus response to a proton pump inhibitor (PPI) trial (esomeprazole 40 mg daily for 2 weeks) among all 308 patients analyzed in the Diamond study ^2^. pH-metry was carried out by using the wireless Bravo system, and a single 24-hour period was analyzed. Studies were interpreted as positive or negative on the basis of both esophageal acid exposure (>5%) and a positive symptom association probability score for their dominant upper gastrointestinal (GI) symptom. A positive response to the PPI test was defined as the absence of the dominant symptom for the last 3 of the 14 days.

The unprecedented therapeutic efficacy of PPIs in healing esophagitis and resolving heartburn spawned the concept of using a short course of high-dose PPIs as a “diagnostic test” for GERD. However, responsiveness to PPIs, abnormal pH-metry, and symptom-based assessments each detect unique patient populations that only partially overlap; a positive (standard-dose) PPI response was observed in 69% of patients with and 51% of patients without endoscopic or pH-metry criteria (or both) for GERD in the Diamond study ( Figure 3) ^3^. Similarly, in a meta-analysis of 15 “PPI test” studies that used pH-metry as the reference standard, the positive likelihood ratio of the “PPI test” for predicting GERD was low, ranging from only 1.63 to 1.87 ^20^.

The imperfect overlap between patient populations defined by physiologic testing and response to a PPI trial does not negate the practicality and cost-effectiveness of empiric PPI therapy. Fass *et al*. calculated that, even though a “PPI test” had only 80% sensitivity and 57% specificity for detecting physiologically defined GERD, this protocol saved an average of $348 per patient by reduction in the use of diagnostic testing ^21^. Nonetheless, empiric PPI therapy has its limitations. A positive response may be attributable to a placebo effect or the presence of an alternative acid-peptic disorder, whereas a negative response may occur with symptoms refractory to PPI therapy ^4^. Another important consideration is the potential to foster unnecessary long-term PPI use, which has clinical and economic implications. In summary, empiric PPI therapy is a simple and cost-effective way to manage typical reflux symptoms in patients without warning signs, but the effectiveness of the therapy does not equate to a diagnosis of GERD or to a need for long-term PPI therapy.

### Ambulatory gastroesophageal reflux disease testing: pH and pH-impedance monitoring

Ambulatory reflux monitoring can quantify reflux, potentially diagnosing GERD in patients without erosive reflux disease. Conventional (or wireless) pH-metry detects reflux events on the basis of their acidity, whereas pH-metry combined with impedance detects all liquid or gas reflux events or both. Esophageal acid exposure is defined as the percentage of the recording time with esophageal pH of less than 4, and reported upper limits of normal range from 3.9% to 7.2% ^22– 24^. The reported sensitivity and specificity of pH-metry for differentiating control subjects from patients with esophagitis are 77–100% and 85–100%, respectively ^25– 28^.

The yield of both pH-metry and pH-impedance monitoring can be increased by testing the relationship between reflux events and patient-reported symptoms, although in the case of pH-metry this analysis is restricted to acid reflux events. However, the significance of the increased yield of the symptom-reflux relationship is unclear, given that only abnormal acid exposure has been shown to correlate with medical or surgical treatment outcome ^29^. This is even more true in the case of pH-impedance testing where the added yield of detecting “non-acid reflux” with impedance technology is negligible at best ^30^. Similarly, except in unusual circumstances in which the pharmacological effectiveness of PPIs is in question, reflux monitoring studies should be done withholding PPI therapy for a week prior to (and during) the study to best address the question “Does my patient have pathological esophageal acid exposure?” ^31^.

## Risks and benefits of long-term proton pump inhibitor use

PPIs have revolutionized the medical approach to upper GI disorders. Initially developed as a treatment for reflux esophagitis, these potent, well-tolerated inhibitors of gastric acid secretion have subsequently proven effective for a broad range of syndromes attributed to acid reflux, acid secretion, or acid hypersecretion. With these broadened indications came an exponential increase in worldwide PPI use ^32, 33^. Another offshoot of the success with PPIs in resolving reflux esophagitis has been the emergence of the logic in clinical practice that “if some is good, more is better” with respect to PPI dosage and symptom control, often ignoring the possibility that the syndrome in question had only a limited relationship to gastric acid secretion in the first place. This paradigm is especially relevant to suspected “atypical symptoms of GERD”; the observation that *some* cases of chronic laryngitis, cough, or wheezing improve with PPI therapy has led to the practice that *all* cases are being treated with high doses of PPIs for extended periods. Consequently, in less than 30 years, PPIs have evolved from tightly regulated medicines approved for short-term use in healing esophagitis to over-the-counter products advertised on television and billboards and used for a wide array of syndromes in which reflux *may* have a potentiating role. Not surprisingly, PPIs are often ineffective when used in this manner.

Coincident with surging PPI usage, the literature surrounding PPI safety and efficacy is also growing exponentially, making it difficult to differentiate fact from fiction. A recent effort at adding clarity to this issue was led by three Italian scientific societies in collaboration with an impressive collection of expert international reviewers. They performed a systematic literature review of almost 500 papers and published a narrative review on the safety and appropriateness of PPI therapy ^34^. Table 1 summarizes their key messages regarding appropriate long-term PPI use in GERD. Examining this result, one can’t help but reflect back to the Diamond study, specifically Figure 3. What this is suggesting is that—apart from the circumstances of high-grade esophagitis, eosinophilic esophagitis, or Barrett’s esophagus—long-term PPI use is warranted if it renders effective symptom control, regardless of any objective evidence of GERD. On the other hand, they suggest PPI use to be of uncertain benefit if the target symptoms were non-responsive or for “extra-digestive GERD”. Basically, this is advocating using the results of a PPI trial, for typical or atypical symptoms, to ascertain whether or not PPI therapy is appropriate.

**Table 1.** Summary of the conclusions by Scarpignato *et al*. ^34^ regarding the appropriateness of long-term PPI therapy in GERD.

Long-term PPI therapy appropriate	PPI use of uncertain benefit
➢ Healing and maintenance of healed Los Angeles grade C or D erosive esophagitis	➢ PPI non-responsive GERD
➢ PPI-responsive GERD/non-erosive reflux disease	➢ Extra-digestive GERD
➢ Barrett’s esophagus	
➢ PPI-responsive esophageal eosinophilia	

GERD, gastroesophageal reflux disease; PPI, proton pump inhibitor.

Coupled with skyrocketing PPI usage has been unprecedented scrutiny of the safety of long-term use and a growing list of associated safety concerns. At and prior to approval, concerns related to chronic PPI therapy centered on consequences of pharmacologically induced hypochlorhydria: hypergastrinemia, gastric cancer, gastric carcinoid tumors, loss of gastric sterility, and micronutrient malabsorption. Hypergastrinemia and increased bacterial colonization of the stomach can be experimentally demonstrated, but there have been no instances of gastric cancers, esophageal cancers, or carcinoids linked to chronic PPI therapy in humans ^35^. On the other hand, gastric acid does facilitate iron and vitamin B _12_ absorption and long-term PPI use has a dose-dependent effect on clinical iron and B _12_ deficiency ^36, 37^. Hypochlorhydria also interferes with the stomach’s bactericidal function, and long-term users are more prone to enteric infections, including *Clostridium difficile* (up to three-fold increase), *Campylobacter*, *Salmonella* (two- to six-fold increase), and small intestinal bacterial overgrowth (two- to eight-fold increase) ^38^. Conversely, despite intense scrutiny for more than ten years, evidence does not support clinically relevant calcium malabsorption or an increased risk of community-acquired pneumonia with chronic PPI use ^38^.

Mass population exposure to PPIs has also revealed potential idiosyncratic reactions. An observational case-control study reported a five-fold increased risk of acute interstitial nephritis among PPI users ^39^. Rare isolated cases of profound PPI-associated hypomagnesemia have also been reported ^40^. However, in neither case is the mechanism understood, and attempts at linking PPI use with chronic kidney disease or hypomagnesemia in population-based studies have yielded only very low hazard ratios (≤1.5), likely representing noise rather than signal ^40^. Similar weak associations with PPI use have been reported for dementia and myocardial infarction in population-based epidemiology studies or meta-analyses or both ^38, 41^. However, in the case of myocardial infarction, this was also tested in a randomized controlled trial. The Clopidogrel and the Optimization of Gastrointestinal Events Trial (COGENT) randomly assigned patients with an indication for dual anti-platelet therapy to receive clopidogrel and aspirin in combination with either omeprazole or placebo. Not only did the omeprazole group experience significant benefit with respect to reduced GI bleeding ( *P* <0.001) but cardiovascular events were actually marginally less frequent, occurring in 4.9% of the omeprazole group compared with 5.7% in the placebo group (not significant) ^42^. Clearly, observational studies have their limits; these studies are inherently flawed by an inability to establish causality, unmeasured confounders, inaccurately measured confounders, and unaccounted-for biases ^43^. Hence, findings of weak associations should be viewed as hypothesis-generating rather than a cause for public hysteria.

Table 2 summarizes available safety information on long-term PPI use with the concerns grouped by the strength of substantiating data and coupled with risk estimate, proposed mechanism, and significance. In the table, there is little of sufficient concern to alter practice, provided that PPI use is appropriate.

**Table 2.** Potential adverse effects reported to be associated with PPI use stratified by estimate of causality along with proposed mechanism, risk estimate, and graded clinical significance.

Risks with an established causal relationship to PPI use			
Putative risk	Proposed mechanism	Risk estimate/Evidence	Clinical significance
Acute interstitial nephritis	Idiosyncratic, rare	Moderate (OR 5.16), Observational (case-control)	Emphasizes need for valid PPI indication
Fundic gland polyp	Hypergastrinemia	Low (OR 2.45), Systematic review, meta-analysis	Minimal
Hypomagnesemia (severe)	Idiosyncratic, rare	Unable to calculate, Observational (case reports)	Emphasizes need for valid PPI indication
Iron deficiency	Hypochlorhydria, poor absorption	Low (OR 2.49), Observational (case-control)	Minimal; treatable and reversible
SIBO	Hypochlorhydria, loss of gastric sterility	Low (OR 2.28), Meta-analysis	Minimal; treatable and reversible
Vitamin B12 deficiency	Hypochlorhydria, poor absorption	Low (HR 1.83) Systematic review, meta-analysis	Minimal; treatable and reversible
Risks with a weak association with PPI use			
Bone fracture	Hypochlorhydria, poor calcium absorption	Low (OR 2.65), Observational (case-control)	Minimal; standard bone health recommendations
Chronic kidney disease	Not established	Low (HR 1.50), Observational (population-based cohort)	Minimal; evidence is too weak
*Clostridium difficile*–associated diarrhea	Hypochlorhydria, loss of gastric sterility	Low (RR 1.69), Meta-analysis	Minimal; emphasizes need for valid PPI indication
Dementia	Beta-amyloid deposits	Very low (HR 1.44), Observational (prospective cohort)	Minimal; evidence is too weak
Hepatic encephalopathy in patients with cirrhosis	SIBO, bacterial translocation	Low (HR 1.72), Observational (case-control)	Minimal; emphasizes need for valid PPI indication
Spontaneous bacterial peritonitis in patients with cirrhosis	SIBO, bacterial translocation	Low (OR 2.28), Systematic review, meta-analysis	Minimal; emphasizes need for valid PPI indication
Hypothesized risks of PPI use, but not reported or observed			
Community-acquired pneumonia	Loss of acid-mediated gastric sterility, aspiration	Very low (OR 1.49), Systematic review, meta-analysis	Minimal; evidence is too weak
Acute cardiovascular events	Drug-drug interaction with hepatic metabolism of clopidogrel	Not observed (HR 0.99), Randomized controlled trial	Minimal; evidence does not support

HR, hazard ratio; OR, odds ratio; PPI, proton pump inhibitor; RR, relative risk; SIBO, small intestinal bacterial overgrowth.

## Conclusions

GERD is common but being more exact than that is difficult because of inconsistencies in how GERD is defined. In clinical practice, the diagnosis is commonly based on a symptom assessment without testing, and, as a general rule, the extent of diagnostic testing should be limited to tests which guide management decisions, detect alternate diagnoses and/or protect the patient from the risk of an inappropriate treatment. The management approach used varies depending on an assessment of the risk that complications exist, the history and success of treatment trials, whether or not a potentially morbid therapy such as anti-reflux surgery is under consideration, and the history of prior testing. When testing is pursued, upper GI endoscopy is the most useful initial diagnostic test because it evaluates for the major potential morbidities (Barrett’s, stricture, and cancer) associated with GERD and allows for the exclusion of some alternative diagnostic possibilities. However, endoscopy is insensitive for diagnosing GERD because most patients with GERD have NERD, which remains a diagnostic dilemma. Although many studies have tried to objectify the diagnosis of GERD with improved technology, this is ultimately a pragmatic diagnosis based on response to PPI therapy, and, in the end, response to PPI therapy becomes the major indication for continued PPI therapy. Conversely, in the absence of objective criteria for GERD and of apparent clinical benefit, PPI therapy is not indicated and should be discontinued. PPIs are well tolerated and safe, but nothing is perfectly safe, and in the absence of measurable clinical benefit, even a miniscule risk dominates the risk-benefit assessment.
